# Assessment of Cystamine’s Radioprotective/Antioxidant Ability under High-Dose-Rate Irradiation: A Monte Carlo Multi-Track Chemistry Simulation Study

**DOI:** 10.3390/antiox12030776

**Published:** 2023-03-22

**Authors:** Samafou Penabeï, Jintana Meesungnoen, Jean-Paul Jay-Gerin

**Affiliations:** Département de Médecine Nucléaire et de Radiobiologie, Faculté de Médecine et des Sciences de la Santé, Université de Sherbrooke, 3001, 12^ème^ Avenue Nord, Sherbrooke, QC J1H 5N4, Canada; samafou.penabei@usherbrooke.ca (S.P.); jintana.meesungnoen@usherbrooke.ca (J.M.)

**Keywords:** cystamine, radioprotector, antioxidant, ferrous sulfate (Fricke) dosimeter, water radiolysis, dose rate, Monte Carlo multi-track chemistry simulation, radiation chemical yields (*G* values), FLASH radiotherapy

## Abstract

(1) Background: cystamine and its reduced form cysteamine have radioprotective/antioxidant effects in vivo. In this study, we use an in vitro model system to examine the behavior of cystamine towards the reactive primary species produced during the radiolysis of the Fricke dosimeter under high dose-rate irradiation conditions. (2) Methods: our approach was to use the familiar radiolytic oxidation of ferrous to ferric ions as an indicator of the radioprotective/antioxidant capacity of cystamine. A Monte Carlo computer code was used to simulate the multi-track radiation-induced chemistry of aerated and deaerated Fricke-cystamine solutions as a function of dose rate while covering a large range of cystamine concentrations. (3) Results: our simulations revealed that cystamine provides better protection at pulsed dose rates compared to conventional, low-dose-rate irradiations. Furthermore, our simulations confirmed the radical-capturing ability of cystamine, clearly indicating the strong antioxidant profile of this compound. (4) Conclusion: assuming that these findings can be transposable to cells and tissues at physiological pH, it is suggested that combining cystamine with FLASH-RT could be a promising approach to further enhance the therapeutic ratio of cancer cure.

## 1. Introduction

The biological effects of radiation on healthy organs surrounding tumour target volumes are a fundamental dose-limiting restriction in radiotherapy (RT). To protect healthy organs from ionizing radiation, and to reduce patient morbidity or mortality, various radioprotectors have been used [1,2]. The clinical involvements of these radioprotective agents have emerged as promising medications with antitumor effects. However, conventional radiotherapy treatments and cures are still limited by acute or chronic toxicities to normal tissue [1,2,3,4,5,6]. Recently, a fundamentally different paradigm of radiation therapy based on delivering radiation at ultra-high dose rates has emerged. This new technique (termed ‘FLASH-RT’) demonstrates a sparing effect on healthy tissues without compromising the anti-tumour action [7,8]. Although FLASH-RT appears to significantly improve the therapeutic ratio of cancer treatment, the protection of surrounding healthy tissue has nevertheless not been shown to be complete. It would therefore be expected that combining a radioprotective agent with FLASH-RT would further improve the therapeutic ratio of cancer cure.

In addition, the potential use of radioprotective agents that can protect large populations in the event of unwanted or unexpected exposures to high-dose-rate radiation (such as severe nuclear plant accidents, nuclear weapons in wartime, nuclear or radiological terrorism) or even astronauts (in deep-space exploration missions) is limited, mainly due to their adverse side-effects. Therefore, it is of great interest to pursue research on currently available radioprotective and radio-mitigating agents as well as the development of new, more efficient, and as far as possible non-toxic ones that could be used immediately when such events occur [9,10].

Water being the most abundant component in living cells and tissues, a detailed knowledge of the radiation chemistry of aqueous solutions is essential to better understand the early stages of the complex chain of radiobiological events that occur after the passage of radiation [11,12]. Following water radiolysis, highly reactive chemical species are generated. These radiolytic species can potentially attack targeted biomacromolecules such as DNA and are largely responsible for subsequent chemical damage [2,12,13]. On a quantitative basis, the precursor species produced in the radiolytic decomposition of pure aerated water are the hydrated electron (e^−^_aq_), H^•^, H_2_, ^•^OH, H_2_O_2_, H_3_O^+^, OH^−^, hydroperoxyl/superoxide (HO_2_^•^/O_2_^•−^) radicals, etc. [14,15,16,17,18]. Under ordinary irradiation conditions (i.e., in the absence of dose-rate effects), there is no overlap between radiation tracks, and the chemical effects of the irradiation can be represented as a sum of the separate effects of individual tracks, which develop independently over time [19]. In this case, the radiation quality (a measure of which is given by the ‘linear energy transfer’ or LET, expressed in keV/μm) is then considered as the main factor controlling the radiation-chemical yields (or *G* values) [4,20,21]. When the radiation dose rate increases, the overall physicochemical and spatiotemporal situation changes significantly as a result of the overlap between adjacent radiation tracks. In this case, there is increased inter-track chemistry [19]. The ionization density gets higher, which promotes radical-radical combination or recombination reactions throughout the solution as radiation tracks develop. It follows that the yield of free radicals decreases with increasing dose rate, while the yield of molecular products increases.

At the molecular level, chemical (i.e., non-biological) radioprotectors are believed to exert their protective effects in cellular systems through a variety of mechanisms, such as free-radical scavenging and hydrogen atom donation [12,22]. The harmful effects of radiation are reduced if the reactive intermediates created by water radiolysis are scavenged (i.e., intercepted) by the protective compound before being able to interact with vital cellular components (particularly DNA) [4]. The cytoprotective action of radioprotective compounds containing sulfhydryl –SH groups (i.e., with labile H^•^ atoms) can also be obtained by donating H^•^ atoms to chemically repair the ‘direct’ or ‘indirect’ lesions in the target biomolecules after they have occurred, but before making the damage permanent by oxygen addition. In the latter scenario, sulfhydryl molecules exert their protective activity by effectively competing with O_2_ in reactions with DNA free radicals [23], thereby minimizing DNA damage and increasing cell survival.

Cystamine is an organic diamino-disulfide compound (RSSR, R = NH_2_–CH_2_–CH_2_) obtained by the oxidative dimerization of cysteamine (RSH), an aminothiol belonging to the same family, well known for its radioprotective properties [24]. In the reducing environment of cells, cystamine is rapidly metabolized into two cysteamine molecules following the cleavage of its very unstable disulfide bond [25]. Current understanding of the mechanism of action of cystamine in vivo [26,27] suggests that cysteamine is the key metabolite responsible for the radiation-protective properties of this compound.

Below pH 8, cystamine occurs mainly in the form of the doubly protonated molecule ^+^NH_3_–CH_2_–CH_2_–S–S–CH_2_–CH_2_–NH_3_^+^ (p*K*_a_~9 for both –NH_3_^+^ groups) [4,6]. The mutual Coulomb repulsion of the two positive charges at opposite ends promotes an open conformation with high collisional accessibility of the S–S center to approaching radicals. This property is an important determinant of this compound’s capacity as a water-based free-radical (such as ^•^OH and H^•^ [28]) scavenger, which in turn explains its strong antioxidant profile.

Several previous studies [29,30] including ours [4,6,31] have used the well-known radiolytic oxidation of ferrous (Fe^2+^) to ferric (Fe^3+^) ions in the aqueous ferrous sulfate, or Fricke, chemical dosimeter [15,32,33] to quantify the radical-scavenging properties of cystamine and thereby evaluate its radioprotective/antioxidant capacity. Although the Fricke dosimeter was originally developed as a dose-measuring device, it is also, at the molecular level, a most valuable tool for examining the effect of the addition of any scavenger of the primary chemical species of water radiolysis on the value of the radiolytic ferric ion, or Fricke, yield *G*(Fe^3+^) [6,34,35,36]. In the event that a radioprotective substance such as cystamine is present in irradiated Fricke solutions, the protector compound will competitively react with the products resulting from the radiolysis of water before they can react with Fe^2+^, and *G*(Fe^3+^) will be reduced (i.e., Fe^2+^ will be protected). These earlier studies using Fricke’s dosimeter solution as an indicator solution demonstrated that the protective effect of cystamine relies on its radical-capturing capacity. The observed decrease in *G*(Fe^3+^) in the presence of cystamine during irradiation was further confirmed using Monte Carlo simulations of the radiolysis of Fricke-cystamine solutions in the presence and absence of O_2_ [4,6].

Unlike previous work, which only considered low-dose-rate irradiation, our goal here is to evaluate the effect of cystamine on the oxidation of Fe^2+^ ions to Fe^3+^ in the Fricke dosimeter under conditions of high dose rates, a situation relating to FLASH-RT or even, for example, a nuclear power plant accident. To this end, we used our recently developed multi-track irradiation model [37] in combination with an extended version of our Monte Carlo computer code IONLYS-IRT [4,6] to simulate the radiolysis of aerated and deaerated Fricke-cystamine solutions by single and instantaneous pulses of 300-MeV incident protons—which mimic the low-LET limit of ^60^Co γ rays or fast electrons (LET~0.3 keV/μm)—while covering a wide range of cystamine concentrations (10^−6^–1 M).

Throughout this article, radiation chemical yields are expressed in the traditionally employed units of molecules formed (or consumed) per 100 eV of energy absorbed, as *g*(X) for primary (or ‘escape’) yields and *G*(X) for experimentally measured yields. For conversion into SI units (mol/J): 1 molecule/100 eV ≈ 0.10364 μmol/J [15,16,17,18].

## 2. Materials and Methods

### 2.1. The Ferrous Sulfate (Fricke) Dosimeter

The ferrous sulfate dosimeter, better known as the ‘Fricke dosimeter’ after Hugo Fricke [32], is the most commonly used liquid chemical dosimeter and certainly the best understood of all aqueous chemical systems studied. Thanks to its accuracy, reproducibility, and linearity of its response as a function of dose, it is widely accepted in radiation-chemical work [15,38,39,40]. The ‘standard’ Fricke solution [15,32] is composed of 1 mM ferrous (Fe^2+^) ions in aqueous 0.4 M sulfuric acid (pH~0.46) and is saturated with air (the concentration of O_2_ is ~2.5 × 10^−4^ M). The mechanism for the radiolytic oxidation of Fe^2+^ to Fe^3+^ ions by the oxidizing species (^•^OH, HO_2_^•^—given the rapid conversion of e^−^_aq_ to H^•^ at low pH, and to HO_2_^•^ in the presence of oxygen—and H_2_O_2_) produced during the radiolysis of water is well understood [15,32,41] and the rate constants at room temperature of the individual reactions involved in the reaction mechanism are known [15,32,33,42]. In short, the main reactions for Fe^3+^ ion production in the Fricke dosimeter are [6]:

e^−^_aq_ + H^+^ → H^•^    *k*_1_ = 2.3 × 10^10^ M^−1^ s^−1^ (1)

H^•^ + O_2_ → HO_2_^•^    *k*_2_ = 2.1 × 10^10^ M^−1^ s^−1^ (2)

^•^OH + Fe^2+^ → Fe^3+^ + OH^−^    *k*_3_ = 3.4 × 10^8^ M^−1^ s^−1^ (3)

HO_2_^•^ + Fe^2+^ → Fe^3+^ + HO_2_^−^    *k*_4_ = 7.9 × 10^5^ M^−1^ s^−1^ (4)

HO_2_^−^ + H^+^ → H_2_O_2_    *k*_5_ = 5.0 × 10^10^ M^−1^ s^−1^ (5)

(6) H2O2+Fe2+→H+Fe3+O•H+OH−  k6 = 52 M−1s−1

H^•^ + Fe^2+^ → Fe^3+^ + H_2_    *k*_7_ = 1.3 × 10^7^ M^−1^ s^−1^, (7)

where the rate constants (*k*) given here for the reactions between ions are at infinite dilution of ions or zero ionic strength.

Considering all sources of Fe^3+^ ions, the Fricke *G*-value in the presence of O_2_ can be expressed in terms of the primary yields of radical and molecular species of the radiolysis of the solution by the stoichiometric equation:

*G*(Fe^3+^)_aerated_ = *g*(^•^OH) + 3 *g*(e^−^_aq_ + H^•^) + 2 *g*(H_2_O_2_) + 3 *g*(HO_2_^•^), (8)

where *g*(^•^OH) = 2.90, *g*(e^−^_aq_ + H^•^) = 3.70 is the sum of the primary yields of e^−^_aq_ and H^•^, *g*(H_2_O_2_) = 0.80, and *g*(HO_2_^•^) = 0.02 [17]. Using these primary yield values in Equation (8) gives a value of *G*(Fe^3+^)_aerated_ which is well within the range of 1 to 2% of the experimentally observed Fe^3+^ ion yield of 15.5 ± 0.2 ions/100 eV for ^60^Co γ-rays [15,32,38,39,40,43].

In the absence of O_2_, H^•^ can no longer react with oxygen and acts as an oxidant with Fe^2+^. As a result, reaction (7) replaces reaction (2) and H^•^ oxidizes only one Fe^2+^ ion instead of three in an aerated solution. In this case, the Fricke *G*-value is given by:

*G*(Fe^3+^)_deaerated_ = *g*(^•^OH) + *g*(e^−^_aq_ + H^•^) + 2 *g*(H_2_O_2_) + 3 *g*(HO_2_^•^), (9)

where the experimentally observed *G*(Fe^3+^)_deaerated_ value is 8.2 ± 0.3 ions/100 eV for ^60^Co γ-radiation [15,32,41,43].

Equations (8) and (9) show that the formation of Fe^3+^ ions is very sensitive to factors that can modify the primary radical yields. Experimentally, in the presence or absence of oxygen, the presence of cystamine in γ-irradiated Fricke solution significantly reduces ferric ion yields. As shown previously [4,6,29,30,31], this decrease in the Fricke *G*-value as a function of cystamine concentration is a clear sign of the scavenging of these radicals by cystamine, which is known to react rapidly with e^−^_aq_, H^•^, and ^•^OH (see below).

### 2.2. The ‘Instantaneous Pulse’ (Dirac) Model for Determining High-Dose-Rate Effects in Water Radiolysis and Aqueous Solutions

**Figure 1:**
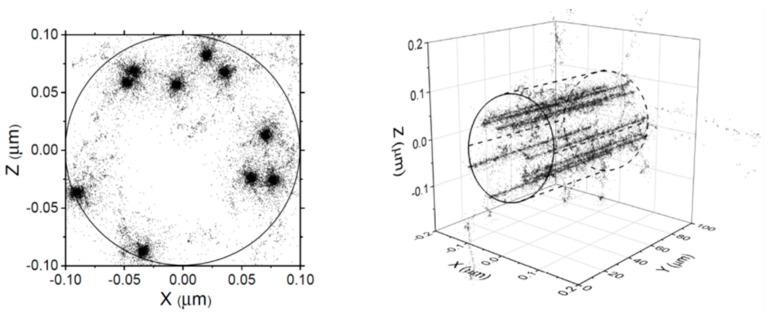
Irradiation model used in this work. (**Right panel**): a three-dimensional representation of a pulse of ten 300-MeV incident protons traversing through the solution calculated at ~1 picosecond (ps) from our Monte Carlo multi-track simulation code. All protons travel along the *Y*-axis over the whole track length used in the simulations (~100 μm). (**Left panel**): the figure shows a front view of the projections within a circle of radius *R*_o_ = 0.1 μm on the *XZ* plane (surface of the solution) for each track segment considered. Note the nonlinear track structure of the δ-rays that result from the knock-on collisions of the incident protons on water. Each dot marks the location of a reactive species. Adapted from Alanazi et al. [37].

We used a multi-track irradiation model [37], recently developed in our laboratory, to study the effects of high dose rates on low-LET water radiolysis at ambient [37,44,45] and elevated [46] temperatures. Briefly, this model consists of randomly irradiating water with single and instantaneous pulses of *N* 300-MeV incident protons which simultaneously penetrate this water perpendicularly inside the surface of a circle of a given radius (see Figure 1). This corresponds to the instantaneous pulse (Dirac) model, in which the pulse duration is assumed to be zero [40]. In this model, all the chemical species are created instantaneously. Any effect due to a finite duration of the pulse or to the interaction between successive pulses is thus neglected. In this case, the absorbed dose per pulse is the only relevant parameter [40].

The advantage of using fast protons (or any other accelerated heavy ion) is that their trajectories are essentially rectilinear [47], therefore, in the case studied in Figure 1, we can define a cylindrical geometry of the beam at the time of entry over the entire track length chosen for the simulations. In this geometry, the proton tracks are all parallel to the cylinder’s axis. Because this cylindrical volume is embedded in non-irradiated bulk water, the radiolytic species which initially form there are not restricted to this volume, but diffuse throughout the solution (infinite in fact) over time. In the end, this situation turns out to be essentially similar to the one we had to deal with previously in our Monte Carlo simulations of the radiolysis of water, including that of Fricke-cystamine solutions (in the absence of dose-rate effects) [4,6,21,48], except that here, instead of simulating a single-proton track at a time, *N* interactive tracks are simulated simultaneously.

Under such conditions, the effect of dose rate was studied by simply varying *N*, the number of incident protons per pulse. In the present work, *N* was chosen to vary from 1 to 1000. Data for *N* = 1, indicating the absence of dose-rate effects, were used as a reference. According to our previous calibration of *N* in terms of dose rate (in Gy/s) (see Figure 3B of Alanazi et al. [37]), the values of *N* between 20 and 1000 are equivalent, under the same irradiation conditions, to dose rates in the range of ~10^7^–4 × 10^9^ Gy/s, respectively. Finally, we chose as time zero the time at which the *N* incident protons reach the front of the cylinder simultaneously.

### 2.3. Radiolysis of Fricke-Cystamine Solutions at High Dose Rates: Monte Carlo Multi-Track Chemistry Simulations

In order to simulate the high-dose-rate radiolysis of the studied aerated and deaerated Fricke-cystamine solutions at 25 °C by 300-MeV irradiating protons, we used an extended version of our Monte Carlo computer code IONLYS-IRT [21,49,50]. Since this version has been described in detail elsewhere [37,44,45,46], only a brief overview of its main features is given below.

In its extended version, our IONLYS ‘step-by-step’ program first models the early spatiotemporal development of *N* incident, interactive proton tracks simultaneously up to ~1 ps. This program is actually composed of two modules: TRACPRO, designed for the transport of the protons under study, and TRACELE, designed for the transport of all secondary electrons (or δ-rays) resulting from the ionization of water molecules. The complex and highly non-homogeneous spatial distribution of reactive species at the end of the physical and physicochemical stages, namely, e^−^_aq_, H^•^, H_2_, ^•^OH, H_2_O_2_, H_3_O^+^, OH^−^, HO_2_^•^, or O_2_^•−^ (p*K*_a_~4.8), O(^1^*D*) and ^•^O^•^(^3^*P*) (oxygen atoms in their singlet ^1^*D* excited state and triplet ^3^*P* ground state, respectively), O^•−^, etc. [21], provided by this program, is subsequently used as the starting point for the chemical stage.

During this third stage of radiation action (>1 ps), the different radiolytic species diffuse away from the site where they originally formed at rates determined by their diffusion coefficients, and react with themselves or in competition (scavenging reaction) with dissolved, uniformly distributed solutes (oxygen in aerated Fricke solution and cystamine in the case of interest here) that are present at the irradiation time. This stage is covered by our IRT program, which employs the ‘independent reaction times’ (IRT) method [51,52,53], an efficient stochastic simulation technique based on the approximation that the reaction time of each pair of reactants is independent of the presence of other particles in the solution. Its detailed implementation has been given previously [37,50]. The ability of this program to provide reliable chemical yields as a function of time has been well validated over a wide range of irradiation conditions by comparison with full random flight Monte Carlo simulations in which the trajectories of diffusing reactive species are closely followed [54,55]. In addition, our IRT program can also effectively describe reactions that take place over long periods of time when tracks no longer exist and the radiolytic products are homogeneously distributed in the solution. This is the case here for the simulation of the radiolysis of the Fricke dosimeter, where the Fe^3+^ ions are generated at different time points up to ~200 s [4,6,33,42,56].

The chemical reaction scheme, rate constants, and diffusion coefficients of the reactive species used in our IONLYS-IRT code for carrying out the simulation of the radiolysis of Fricke-cystamine solutions were the same as those used previously [4,6]. To summarize, we added to the reaction scheme for the radiolysis of pure liquid water [18,21,33,50] the reactions listed in Table 1 of Autsavapromporn et al. [42], which take into account the species HSO_4_^−^, SO_4_^2−^, SO_4_^•−^ and S_2_O_8_^2−^ present in irradiated 0.4 M H_2_SO_4_ aqueous solutions [33,57]. In order to simulate the chemistry of the Fricke dosimeter, we included in the IRT program the reactions (3), (4), (6), and (7) of Fe^2+^ ions with the various oxidizing species which are formed in the irradiated water. As seen above, in the absence of oxygen, the difference observed in the Fricke yield comes from the replacement of reaction (2) by reaction (7). Moreover, under the irradiation conditions of this study, the concentrations of radiolytic products remained low enough compared to the background concentrations of H^+^ (~0.4 M), Fe^2+^ ions (1 mM), O_2_ (~0.25 mM), and cystamine (up to 1 M) in a solution that their reactions could be treated in the IRT program as the pseudo-first order.

Finally, in order to simulate the radiolysis of aerated or deaerated Fricke-cystamine (RSSR) solutions, we extended the reaction scheme for the Fricke dosimeter to include the 27 chemical reactions listed in Table 2 of Meesat et al. [6]. Of these reactions, the most important for the production of Fe^3+^ are [4,6]:

RSSR + e^−^_aq_ → (RSSR)^• −^    *k*_10_ = 4.1 × 10^10^ M^−1^ s^−1^ (10)

RSSR + H^•^ → RS^•^ + RSH    *k*_11_ = 8 × 10^9^ M^−1^ s^−1^ (11)

RSSR + ^•^OH → (RSSR)^•+^ + OH^−^    *k*_12_ = 1.7 × 10^10^ M^−1^ s^−1^ (12)

Fe^2+^ + RS^•^ → Fe^3+^ + RS^−^    *k*_13_ = 2.5 × 10^8^ M^−1^ s^−1^ (13)

Fe^2+^ + (RSSR)^•+^ → Fe^3+^ + RSSR    *k*_14_ = 2 × 10^6^ M^−1^ s^−1^ (14)

RS^•^ + RSSR → RSSSR + R^•^    *k*_15_ = 10^6^ M^−1^ s^−1^ (15)

R^•^ + O_2_ → ROO^•^    *k*_16_ = 2 × 10^9^ M^−1^ s^−1^ (16)

(17) $${\mathrm{Fe}}^{2+}\;+\;ROO^{•}\begin{array}{c} H^{+}\\ \to \end{array}Fe^{3}\;+\;\mathrm{ROOH}\;k_{17}\;=\;7.9\times 10^{5}\;M^{-1}s^{-1}$$

(RSSR)^•+^ + (RSSR)^•+^ → (RSSR)^2+^ + RSSR    *k*_18_ = 2.5 × 10^9^ M^−1^ s^−1^, (18)

where the rate constants quoted here for reactions between ions are at infinite dilution (i.e., when no ion-ion interactions occur). In fact, in the IRT program, we considered the effect of the ionic strength of the solutions for all reactions between ions, with the only exception being the self-recombination of e^−^_aq_ for which there is no evidence of any ionic strength effect [58]. Correction of reaction rate constants for the ionic strength was performed using the same procedure as previously used by Meesat et al. [6].

In addition, we also neglected the contribution of the ‘direct’ action of ionizing radiation on the various solutes present in the solution. This is a reasonable approximation judging from the range of H_2_SO_4_, ferrous ions, dissolved oxygen, and cystamine concentrations considered [4,6].

All calculations were performed by simulating short (typically ~5–150 μm, depending on *N*) track segments of 300-MeV irradiating protons. The energy and the LET (~0.3 keV/μm) of the protons remained nearly constant over these simulated track segments. Under our irradiation conditions, the number of simulated ‘histories’ (i.e., the number of pulses, usually 5–100, depending on the value of *N* considered) was chosen to ensure only small statistical fluctuations when calculating average chemical yields while keeping acceptable computer time limits.

## 3. Results and Discussion

**Figure 2:**
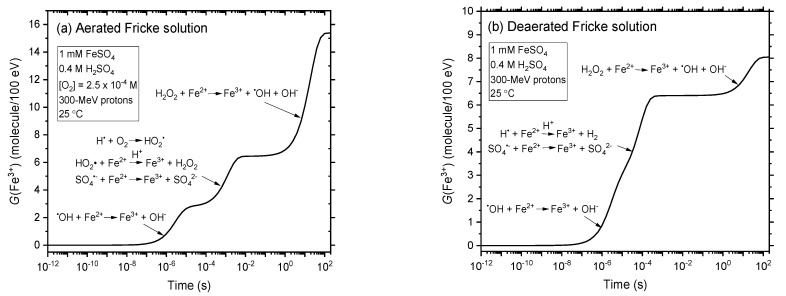
Temporal evolution of *G*(Fe^3+^) obtained from our Monte Carlo simulations of the radiolysis of the Fricke solution (1 mM Fe^2+^ ions in aqueous 0.4 M H_2_SO_4_) under aerated (panel (**a**)) and deaerated (panel (**b**)) conditions, using 300-MeV incident protons at 25 °C, in the interval of ~1 ps–200 s. Data are for *N* = 1 (i.e., in the absence of dose-rate effects); they mimic the Fricke dosimeter radiolysis by ^60^Co γ-rays or fast electrons. Note here that, for solutions of 0.4 M in H_2_SO_4_, a small number of ^•^OH radicals react with HSO_4_^−^ to form the sulfate radical SO_4_^•−^. Nevertheless, the overall Fe^3+^ yield remains the same as that given by Equations (8) and (9), SO_4_^•−^ reacting with Fe^2+^ in a similar way as ^•^OH: SO_4_^•−^ + Fe^2+^ → Fe^3+^ + SO_4_^2−^ (*k* = 9.9 × 10^8^ M^−1^ s^−1^) [6].

Figure 2a,b shows the time evolution of *G*(Fe^3+^) obtained from our simulations of the radiolysis of the Fricke dosimeter under aerated and deaerated conditions, respectively, for incident protons of 300 MeV at 25 °C in the interval of ~1 ps–200 s, in the absence of dose-rate effects (i.e., for *N* = 1). We note that our computed values of *G*(Fe^3+^) (~15.4 and 8.05 molecules/100 eV for aerated and deaerated solutions, respectively) agree very well with the recommended values of 15.5 ± 0.2 and 8.2 ± 0.3 molecules/100 eV for the corresponding Fe^3+^ ion yields in the Fricke dosimeter in the presence or absence of O_2_ for ^60^Co γ-rays or fast electrons [15,32,38,39,40,41,43]. As can be seen, *G*(Fe^3+^) is time-dependent, a consequence of the differences in the time scales of the reactions of Fe^2+^ with the various species created by radiolysis of acidic water (namely, ^•^OH, HO_2_^•^ or H^•^, SO_4_^•−^ and H_2_O_2_) under aerated or deaerated conditions. For instance, the fastest reaction of Fe^2+^ is with ^•^OH while the slowest is with H_2_O_2_. The kinetics of Fe^3+^ formation in the Fricke dosimeter has already been extensively detailed previously [4,6,33,42,56,59] and we will not dwell on it further here.

**Figure 3:**
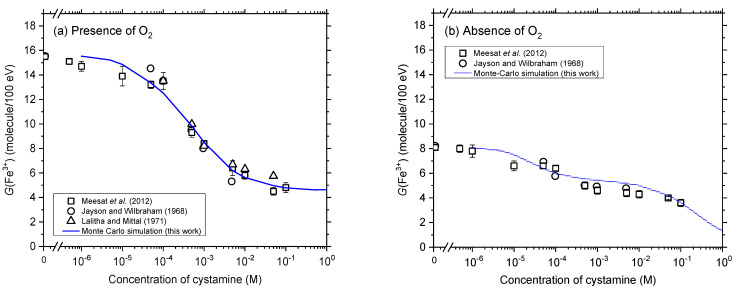
Dependence of the yield of Fe^3+^ ions obtained from our Monte Carlo simulations of the radiolysis of Fricke-cystamine solutions (at ~200 s after ionization) upon the concentration of added cystamine in the range 10^−6^–1 M, using 300-MeV incident protons in the absence of dose-rate effects (*N* = 1) under aerated (panel (**a**)) and deaerated (panel (**b**)) conditions at 25 °C (solid line). Experiment: (○) Jayson and Wilbraham [29], (Δ) Lalitha and Mittal [30], and (**□**) Meesat et al. [6].

The influence of the concentration of added cystamine on the yield of Fricke is illustrated in Figure 3a,b, where our calculated *G*(Fe^3+^) values are reported for *N* = 1 for Fricke-cystamine solutions in the presence and absence of O_2_, respectively, and for cystamine concentrations ranging from 10^−6^ to 1 M. As can be seen, the addition of cystamine markedly reduces *G*(Fe^3+^) under both aerated and deaerated conditions. As discussed in detail previously [4,6], this decrease in *G*(Fe^3+^) indicates that cystamine can easily remove the radiolytic species capable of predominantly attacking Fe^2+^ ions in acidic solution, namely, H^•^ atoms and ^•^OH radicals [reactions (11) and (12)]. This radical-capturing capacity of cystamine readily explains the radiation-protective (antioxidant) profile of this compound. Confirming our previous studies [4,6,31], Figure 3a,b show that our calculated *G*(Fe^3+^) values reproduce very well, without using any free adjustable parameters, the yields of Fe^3+^ ions reported experimentally for X- and ^60^Co γ-irradiations [6,29,30]. Such quantitative agreement between simulated and experimental *G*(Fe^3+^) values is important because it supports the validity of the overall reaction scheme adopted in this work to describe the radiation chemistry of cystamine in aerated and deaerated Fricke solutions.

**Figure 4:**
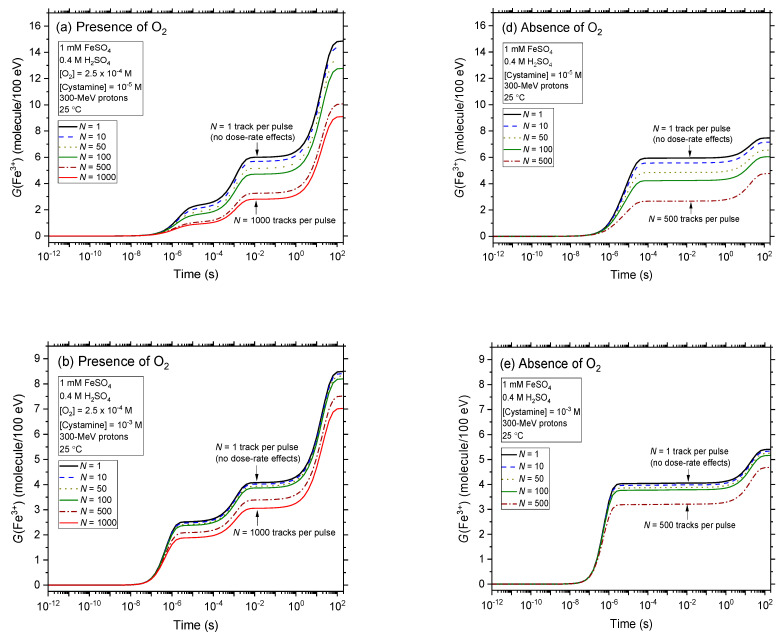

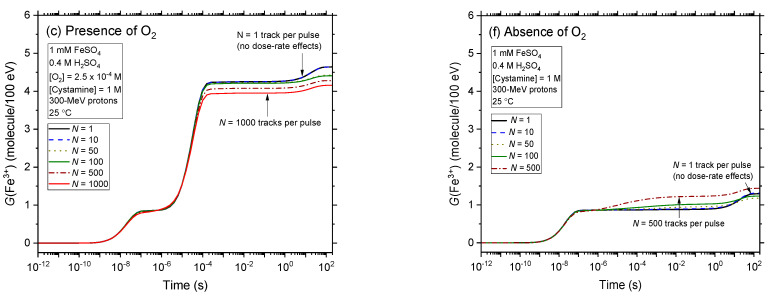
Effect of dose rate (described by *N*, the number of irradiating protons per pulse) on the time evolution of *G*(Fe^3+^) obtained from our Monte Carlo simulations of the radiolysis of aerated (panels (**a**–**c**)) and deaerated (panels (**d**–**f**)) Fricke-cystamine solutions containing various concentrations of cystamine (10^−5^, 10^−3^, and 1 M), using 300-MeV incident protons at 25 °C. Data for *N* = 1 corresponds to the absence of dose-rate effects and are used as a reference. For clarity, only the curves for *N* = 1, 10, 50, 100, 500, and 1000 are shown here.

In Figure 4a–f, we compare the effect of dose rate (described by *N*, the number of proton tracks per pulse) on the kinetics of Fe^3+^ formation, as obtained from our simulations of the radiolysis of aerated and deaerated Fricke-cystamine solutions for a few values of *N* chosen as examples between 1 and 1000 and in the presence of various cystamine concentrations (10^−5^, 10^−3^, and 1 M). As can be seen, *G*(Fe^3+^) decreases markedly with increasing cystamine concentration under both aerated and deaerated conditions for all *N* values. For example, in the absence of dose rate effects (*N* = 1), for air-saturated solutions, *G*(Fe^3+^) decreases from ~14.9 to 4.6 ions per 100 eV (i.e., a ~10.2 *G*-unit decrease) as the concentration of cystamine increases from 10^−5^ to 1 M. However, this decrease of *G*(Fe^3+^) is greatly attenuated as the dose rate increases; indeed, for *N* = 1000 in the presence of oxygen, *G*(Fe^3+^) goes from 9.1 to 4.2 ions per 100 eV (i.e., a reduction of 4.9 *G*-units) between 10^−5^ and 1 M cystamine. Even if the reaction scheme differs significantly, the deaerated solutions show a relatively similar variation of *G*(Fe^3+^) with the concentration of cystamine, with the difference, however, that the drop in the ferric ion yield, when going from 10^−3^ to 1 M cystamine, is more pronounced, whatever the value is chosen for *N*.

**Figure 5:**
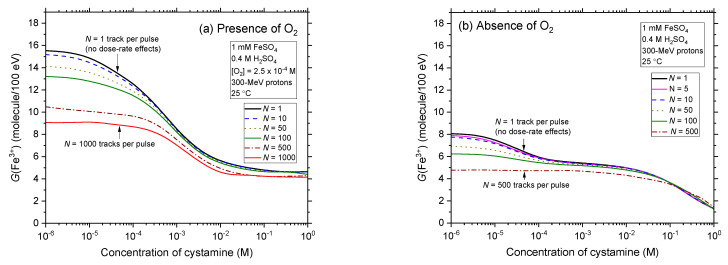
Dependence of Fe^3+^ ion production from irradiated Fricke-cystamine solutions upon the concentration of added cystamine in the range from 10^−6^ to 1 M for different values of *N* under aerated (panel (**a**)) and deaerated (panel (**b**)) conditions. Data for *N* = 1 (absence of dose-rate effects) are used as a reference. As before, for the sake of clarity, we only show here the curves for *N* = 1, 10, 50, 100, 500, and 1000.

The effect of dose rate on the variation of the Fricke yield with the concentration of cystamine is further illustrated in Figure 5a,b over the range of 10^−6^–1 M, for aerated and deaerated Fricke-cystamine solutions, respectively. As can be seen, as *N* increases from 1 to 1000, *G*(Fe^3+^) in aerated solutions gradually decreases at low cystamine concentrations, eventually reaching a value of about ~9 ions per 100 eV for *N* = 1000. In deaerated solutions, this decrease is also significant in this same cystamine concentration range. At these low concentrations, even if *G*(Fe^3+^) decreases, cystamine is less and less active as the dose rate increases. For instance, for *N* = 1000, Figure 5a shows that *G*(Fe^3+^) is more or less independent of the cystamine concentration below, say, ~0.5 mM. This is easily explained by the fact that at high dose rates, the higher concentration of reactants for denser ionizing radiations favors fast intertrack radical-radical combination and recombination reactions in the tracking stage of radiolysis. This leads to the production of fewer and fewer radicals (such as those with which cystamine reacts) and more and more molecular products such as H_2_O_2_, H_2_, or reformed water, which are very unreactive towards cystamine. It can therefore be said that the marked decrease in *G*(Fe^3+^) observed in Figure 5 at low concentrations of cystamine results from two additive radioprotective actions: that of the effect of the dose rate itself and due to the presence of cystamine. At high values of *N*, the dose-rate effect predominates.

In contrast, at concentrations greater than ~0.5 mM, *G*(Fe^3+^) begins to decline sharply again with increasing cystamine concentration. As just discussed above, this decline of *G*(Fe^3+^) shows that at these concentrations it is the effect of the presence of cystamine, rather than the dose rate itself, that predominates the observed radioprotection of Fe^2+^ ions in the radiolysis of Fricke-cystamine solutions at high dose rates.

Finally, Figure 5a,b clearly shows that all the curves of *G*(Fe^3+^) as a function of the concentration of cystamine obtained for *N* > 1 remain lower than that obtained in the absence of dose-rate effects (*N* = 1). Our simulations thus reveal that the addition of cystamine offers a protective effect towards the Fricke dosimeter solution greater at high dose rates than that observed in the absence of dose-rate effects. Assuming that this differential protective role of cystamine is transposable to biological systems (i.e., at physiological pH ~7.4, instead of 0.46 for the Fricke solution), these results would suggest that cystamine may provide increased protection of normal (aerated) tissue at pulsed (FLASH) dose rates (high *N* values) compared to the low dose rates (*N* = 1) delivered in conventional RT irradiations. In other words, a combination of cystamine with FLASH-RT would be expected to act additively, thus offering a promising approach to further improve the therapeutic ratio of cancer cure.

## 4. Conclusions

In this work, Monte Carlo multi-track chemistry simulations of the radiolysis of aerated and deaerated Fricke-cystamine solutions at 25 °C were used in combination with a cylindrical ‘instantaneous pulse’ (Dirac) model in order to quantitatively assess the radioprotective/antioxidant capacity of cystamine under (very) high-dose-rate irradiation conditions. For this, we examined from a purely radiation-chemical perspective the behavior of this compound with respect to the primary chemical species produced in the radiolysis of the Fricke (ferrous sulfate) dosimeter by *N* interactive tracks of 300-MeV irradiating protons, which mimic the low-LET limit of ^60^Co γ-rays or fast electrons (LET~0.3 keV/μm). The effect of dose rate was studied by varying *N*, the ‘number of incident protons per pulse’. The well-known radiolytic oxidation of Fe^2+^ ions to Fe^3+^ was used as an indicator and formed the basis of our method.

The results obtained in this work clearly showed that the protecting/antioxidant effect of cystamine toward the Fricke solution came from its radical-scavenging capacity, which allows this compound to act in competition with the Fe^2+^ ions for the ^•^OH and H^•^ free radicals that result from the radiolysis of acidic water.

A noteworthy result of our simulations is that the addition of cystamine offers a protective effect towards the Fricke dosimeter solution greater at high dose rates than that observed in the absence of dose rate effects. Based on such results and assuming that they can be transposable to biological systems (at physiological pH), it would then appear that cystamine could provide enhanced protection of normal (aerated) tissue at pulsed (FLASH) dose rates compared to low dose rates such as those used in conventional RT irradiations. Under these conditions, combining cystamine with FLASH-RT should act additively, thus offering a promising approach to further improve the therapeutic ratio of cancer cure.

The findings of this work are of evident interest in terms of predictability. Nevertheless, the high consistency between the calculated and measured yield values under low-dose-rate irradiation conditions supports the computational approach and its relevance for understanding, at the molecular level, the indirect radiation damage caused by high-dose-rate irradiation to complex molecules such as cystamine whose radiolysis has never been previously investigated using Monte Carlo multi-track chemistry simulations. We believe that this basic research will be of interest to clinicians working in the field of proton FLASH radiotherapy, as well as for the protection of the public in the event of large-scale radiation exposures at high dose rates.

## Data Availability

Data generated or analyzed during this study are provided in full within the article.
